# The Role of RAC2 and PTTG1 in Cancer Biology

**DOI:** 10.3390/cells14050330

**Published:** 2025-02-23

**Authors:** Katarzyna Rakoczy, Natalia Szymańska, Jakub Stecko, Michał Kisiel, Jakub Sleziak, Agnieszka Gajewska-Naryniecka, Julita Kulbacka

**Affiliations:** 1 Faculty of Medicine, Wroclaw Medical University, Pasteura 1, 50-367 Wroclaw, Poland; katarzyna.rakoczy@student.umw.edu.pl (K.R.); natalia.szymanska@student.umw.edu.pl (N.S.); jakub.stecko@student.umw.edu.pl (J.S.); michal.kisiel@student.umw.edu.pl (M.K.); jakub.sleziak@student.umw.edu.pl (J.S.); 2 Department of Molecular and Cellular Biology, Faculty of Pharmacy, Wroclaw Medical University, Borowska 211a, 50-556 Wroclaw, Poland; agnieszka.gajewska-naryniecka@umw.edu.pl; 3 Department of Immunology and Bioelectrochemistry, State Research Institute Centre for Innovative Medicine, Santariškių g. 5, LT-08406 Vilnius, Lithuania

**Keywords:** cancer, cancer stem cells, RAC2, PTTG1

## Abstract

Several molecular pathways are likely involved in the regulation of cancer stem cells (CSCs) via Ras-associated C3 botulinum toxin substrate 2, RAC2, and pituitary tumor-transforming gene 1 product, PTTG1, given their roles in cellular signaling, survival, proliferation, and metastasis. RAC2 is a member of the Rho GTPase family and plays a crucial role in actin cytoskeleton dynamics, reactive oxygen species production, and cell migration, contributing to epithelial–mesenchymal transition (EMT), immune evasion, and therapy resistance. PTTG1, also known as human securin, regulates key processes such as cell cycle progression, apoptosis suppression, and EMT, promoting metastasis and enhancing cancer cell survival. This article aims to describe the molecular pathways involved in the proliferation, invasiveness, and drug response of cancer cells through RAC2 and PTTG1, aiming to clarify their respective roles in neoplastic process dependencies. Both proteins are involved in critical signaling pathways, including PI3K/AKT, TGF-β, and NF-κB, which facilitate tumor progression by modulating CSC properties, angiogenesis, and immune response. This review highlights the molecular mechanisms by which RAC2 and PTTG1 influence tumorigenesis and describes their potential and efficacy as prognostic biomarkers and therapeutic targets in managing various neoplasms.

## 1. Introduction

Hallmarks of cancer cells, including their replicative immortality, continuous proliferation, angiogenesis, metastasis, and apoptotic reluctance, result from the accumulation of numerous genetic, epigenetic, and transcriptional modifications that cause the transformation of a healthy cell into a neoplastic cell [1]. Even though every neoplasm emerges from a single transformed cell, widely recognized heterogeneity constitutes a common characteristic feature of tumors. Firstly, intertumoral heterogeneity is evident, as tumors manifesting in various patients with a specific type of cancer exhibit significant differences. This heterogeneity is driven by a unique combination of intrinsic and extrinsic factors, including the molecular characteristics of primary cancer cells, their mutational profiles, and the complex conditions of the microenvironment [2]. Secondly, intratumoral heterogeneity occurs within a single specific neoplasm, as cancer cells express different markers as and incorporate varied populations of cells, both proliferative and more differentiated, including cancer stem cells (CSCs) [3]. CSCs constitute the subgroup of cancer cells that exhibits the greatest proliferative potential in contrast to more mature cells, transit amplifying cells, whose self-renewal capacity gradually decreases until they become non-proliferative cancer cells [4]. The concept of CSCs was first demonstrated in human acute leukemia [5], followed by research conducted on solid tumors [6]. Even though there is still no certainty that all types of tumors present in the described the hierarchical system, research on CSCs is being intensively conducted, allowing us to develop increasingly more CSC-based therapeutic approaches. For instance, as in the case of some neoplasms, CSCs express novel markers that are absent in the cells of origin, and targeting these markers allows for lineage ablation. This principle has been demonstrated through the example of doublecortin-like kinase protein 1 (Dclk1), which within a healthy intestinal environment indicates differentiated cells, in contrast to polyps and adenomas, where Dclk1 marks rapidly expanding CSCs that underlie the tumor growth [7]. Dclk1+ cell lineage ablation causes tumor regression without damage to the normal intestine tissue. Targeting CSCs specifically ensures a means to implement effective therapy without impairing the function of normal intestine stem cells [7].

The significant diversity of molecular mechanisms that regulate CSCs and thus underlie tumorigenesis results in ambiguity of the roles that specific genes play in tumor development [3]. For this reason, it is of great importance to thoroughly examine the expression of those genes as well as the pathways that they co-create in order to consider the possibility of their clinical application. In the molecular labyrinth of the cancer cell, several pathways involved in CSCs regulation attract considerable attention. Ras-associated C3 botulinum toxin substrate 2 (RAC2) is a representative of small signaling G proteins, a class of low molecular weight proteins that catalyze the interconversion of GTP to GDP and are located in both the cytoplasm and the inner plasma membrane [8]. RAC proteins belong to the Rho family, the members of which are involved in varied regulatory pathways that include cytoskeleton formation, cell proliferation, and adhesion [9]. RAC2 plays important regulatory roles in actin-based cytoskeleton formation and cell signaling. It is exclusively expressed in blood-derived cells and can be involved in hematopoietic cell formation [10,11]. Research suggests that the RAC2 expression is the highest in bone marrow, lymph nodes, thymus, tonsils, spleen, and, among immune cells, in eosinophils, preceded by neutrophils, basophils, regulatory T cells, and natural killer (NK) cells [12]. Importantly, there is a relationship between altered RAC2 cellular levels and pathological stages of different types of cancers [12], which will be meticulously described in this article. Pituitary tumor-transforming gene 1 (PTTG1) product, also known as human securin, constitutes a versatile protein that is engaged in many cellular pathways, including mitosis regulation, apoptosis, epithelial–mesenchymal transition (EMT), and mitogen-activated protein kinase (MAPK) signaling pathways [13,14]. EMT is a process that occurs naturally in wound repair and inflammation, where epithelial cells lose some of their attributes, including polarity and the ability to adhere, simultaneously gaining novel features, such as enhanced migration and invasion ability. Apart from its positive role, EMT could lead to tumor formation [15]. PTTG1 is connected with various tumors through distinct pathways. As in numerous neoplasms, such as pancreatic cancer [16], prostate cancer [17], lung adenocarcinoma [18], and hepatocellular carcinoma [19], PTTG1 overexpression is connected with tumorigenesis progression and poor prognosis [20]; it constitutes a potential prognostic biomarker as well as a novel target for cancer therapy. This article aims to describe the molecular pathways involved in CSCs regulation via RAC2 and PTTG1 in order to crystallize their roles among neoplastic dependencies.

## 2. RAC2-Driven Pathways

### 2.1. Actin Cytoskeleton Reorganization

The study of C.D. Nobes and A. Hall showed that RAC regulates the polymerization of actin to produce stress fibers and lamellipodia, thus enabling cell movement and migration [20]. Although this study was conducted about 30 years ago, the significance of Rho GTPases in actin cytoskeletal changes is still an important issue in the scientific world. The actin cytoskeleton is fundamental for cellular structure, facilitating movement and other dynamic processes. Its reorganization is a hallmark of cancer cell migration, invasion, and metastasis, particularly in pathways driven by Rho family GTPases [21,22]. RAC2, as well as other Rho family GTPases, is activated by TGF-β and acts in a cofilin-1 phosphorylation via LIMK1/2 [22,23]. Phosphorylated cofilin-1 stabilizes the cytoskeleton by losing its ability to depolymerize actin [22]. Studies on prostate cancer cells proved that targeting the RAC2/PAK4/LIMK1/cofilin pathway impeded cancer progression and reinstated arsenic-induced cancer cell apoptosis [24,25].

### 2.2. Reactive Oxygen Species Production

Rho GTPases RAC1 and RAC2 are part of NADPH oxidase (NOX2) and thus play an important role in the activation and production of reactive oxygen species (ROS) [26]. As RAC2 binds to p67, a conformational change occurs in NOX2 that drives electron transport across the membrane to molecular oxygen-producing superoxide [27]. The study of Nieborowska-Skorska, M. et al. shows that RAC2 regulates ROS generation in primitive chronic myeloid leukemia (CML) cells, which causes oxidative DNA damage and genomic instability, which is a characteristic feature of CSCs [28]. Higher ROS concentrations lead to metabolic disturbance in CSCs as cells lose control over cell cycle and differentiation [29]. CSCs are able to utilize this instability and heterogeneity to modulate the surrounding environment and immune system, as well as evade immune cells [30,31]. ROS accumulation occurs due to RAC2 modifying mitochondrial membrane potential and electron flow via the mitochondrial respiratory chain complex III (MRC-cIII) [28,32]. Down-regulation of RAC2-GTP inhibits levels of mitochondrial superoxide anion (•O2−) and cellular levels of hydrogen peroxide (H_2_O_2_) and highly reactive hydroxyl radical (•OH−) by about 2-fold, which correlates with reduced oxidative DNA damage by twofold [28]. As RAC2 is a subunit of NADPH oxidase, it may influence cellular radiosensitivity and the development of resistance to ionizing radiation [33].

### 2.3. NOTCH Signaling Pathway

NOTCH signaling plays a pivotal role in cancer development [34]. As a primitive signaling pathway involved in multicellular organism development, aberrant NOTCH activation can lead to serious pathological outcomes. The pathway regulates EMT through multiple mechanisms and molecular interactions. NOTCH activation in endothelial cells triggers morphological and phenotypic changes consistent with mesenchymal transformation, including downregulation of epithelial markers like VE-cadherin, TIE1, TIE2, and platelet-endothelial cell adhesion molecule-1 (PECAM-1) as well as upregulation of mesenchymal markers such as α-SMA, platelet-derived growth factor receptors, and fibronectin [34]. NOTCH signaling interfaces with multiple pathways, including PI3K/Akt, cytokine signaling through IL-6/STAT3, and hypoxia response through HIF-1α, to coordinate the EMT program [35,36]. This complex signaling network allows NOTCH to drive invasion, metastasis, and cancer stem cell properties through EMT induction in various cancer types. The relationship between RAC2 and NOTCH signaling appears to be complex and context dependent. In T cell development, RAC2 (along with RAC1) negatively regulates NOTCH signaling, evidenced by increased expression of NOTCH target genes (NOTCHsta3, Hes1, Deltex1, and preTα) in RAC1/RAC2-deficient thymocytes [37,38]. This negative regulation appears to be part of a pathway where RAC proteins help transduce pre-TCR signals that inhibit NOTCH function during early thymic development [38]. However, in chronic lymphocytic leukemia (CLL) cells with NOTCH1 mutations, RAC2 appears to function downstream of NOTCH signaling as inhibition of the NOTCH pathway with PF-03084014 leads to downregulation of RAC2 along with other genes involved in invasion and chemotaxis [39]. This relationship is specifically observed in NOTCH1-mutated cases but not in unmutated cases [39]. These findings suggest that the interaction between RAC2 and NOTCH signaling may be bidirectional and tissue-specific, with RAC2 acting as both a negative regulator of NOTCH in normal T cell development and as a downstream effector of NOTCH signaling in certain leukemic contexts.

### 2.4. PI3K/AKT/mTOR Pathway

The PI3K/AKT/mTOR pathway regulates multiple cellular physiological processes and is associated with certain tumors [40]. Moreover, PI3K up-regulates a group of proteins, called guanine exchange factors (GEFs), required for the activation of RAC2, as GEFs cause GDP dissociation and GTP binding [41,42,43]. The PI3K and RAC2 cooperation is instrumental in chemotactic cell movements, especially in neutrophils, since the first protein enables sensing and polarizing the cell toward a chemoattractant gradient, whereas the second plays a crucial role in cell movement [42,44].

### 2.5. JAK/STAT Pathway

The JAK/STAT signaling pathway is associated with tumor growth, progression, chemoresistance, and the generation of CSCs [45]. When cytokine locally produced by cancer cells binds to its receptor, it triggers JAK activation and mediates intracellular signaling cascades, leading to STATs’ phosphorylation at the tyrosine residue [45,46]. Then, phosphorylated STAT is transported into the nucleus, where it can act as a transcriptional factor [46]. Furthermore, the study of Pelletier, S. et al. proved that RAC GTPases are required for the activation of JAK/STAT signaling by G protein-coupled receptors [47]. However, there is no evidence that RAC2 possesses this particular ability; therefore, further studies are required.

### 2.6. MAPK Pathway

**Figure 1:**
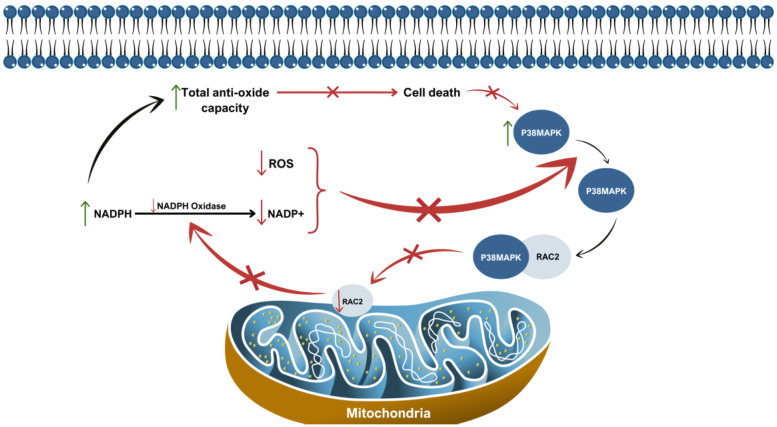
Schematic visualization of the feedback loop between ROS, RAC2, and P38 MAPK that induces G0 cells’ resistance to ionizing radiation [33].

The ERK-MAPK signaling pathway is involved in cell motility and invasion in colon cancer [48]. As mentioned beforehand, RAC is responsible for cell migration through actin cytoskeletal reorganization, and in the field of this feature, RAC can be activated by the ERK-MAPK pathway [48]. In human melanoma cells, the guanine-nucleotide exchange factor (GEF) PREX1, an activator of Rho GTPases, plays a crucial role in facilitating cell invasion. The expression of PREX1 is regulated by the ERK-MAPK pathway [49]. Ultimately, the ERK-MAPK pathway leads to overexpression of PREX1, subsequently activating RAC, thereby enabling the invasive capability of melanoma cells [50]. Moreover, there is a connection between ROS production, RAC2, and P38 MAPK [33]. The study of Pei H. et al. proved the existence of a feedback loop between these three factors, which induces resistance of G0 cells to ionizing radiation [33]. P38 MAPK interacts with RAC2, resulting in the decrease in functional RAC2 and eventually leading to reduced ROS levels, which makes cells more resistant to radiation (Figure 1) [33].

### 2.7. Current Therapeutic RAC2-Related Strategies

RAC2 is a promising target for cancer therapy, although its role seems context-dependent and requires further investigation. Preclinical evidence indicates RAC2 may function as both a prognostic biomarker and a therapeutic target [51]. The analysis of the data from Cancer Genome Atlas Kidney Renal Clear Cell Carcinoma (TCGA-KIRC) showed that RAC2 is significantly upregulated in clear cell renal cell carcinoma (ccRCC). High RAC2 expression is associated with higher clinical and pathological grade and poorer prognosis in patients with clear cell renal cell carcinoma (ccRCC) [51]. The obtained results suggest that RAC2 may serve as a prognostic biomarker in patients with ccRCC and a therapeutic target for treating ccRCC [51].

In contrast, the relationship between RAC2 expression and prognosis in breast cancer appears to be more intricate. While some studies indicate that RAC2 overexpression suppresses MCF-7 cell proliferation [49,52]. In contrast to ccRCC, in BC, a better prognosis was linked to increased expression of RAC2 [49]. This inconsistency highlights the need for additional research to clarify RAC2’s precise role in BC. Chen, Q. et al. proposed a unique pyroptosis-related genes prognostic molecular model, which included APOBEC3D, TNFRSF14, and RAC2, to evaluate prognosis and immune infiltration in BC patients [52]. Additionally, a study by Xu, Y. et al. showed that a high level of RAC2 lowers the protumor/antitumor immune cell ratio [49].

The contrasting roles of RAC2 in ccRCC and BC indicate the need for context-specific research to clarify the exact molecular mechanism by which RAC2 impacts tumor progression in different cancer types. In cancer where overexpression of RAC2 is associated with poor prognosis, strictly targeting RAC2 can be a potential therapeutic strategy. Consequently, additional studies that focus on specific inhibitors of RAC2 or other therapeutic approaches are required. Future studies should investigate the effectiveness of combining RAC2-targeted therapies with existing treatments to overcome resistance and enhance the efficacy of anticancer treatment. Moreover, further research is needed to validate RAC2 as a reliable prognostic biomarker.

## 3. PTTG1-Driven Pathways

### 3.1. Interaction Between PTTG1 and p53

The interaction between PTTG1 and the tumor suppressor p53 is complex and warrants further investigation. While evidence suggests a relationship exists, the nature and implications of this interaction remain incompletely understood [53]. Studies have revealed a feedback loop between PTTG1-targeting miRNAs, PTTG1, and p53 in pituitary tumorigenesis [54]. On the contrary, the study of Noll, J.E. et al. on multiple myeloma plasma cells shows no correlation between PTTG1 and p53 expression, highlighting the context-dependent nature of this interaction [55]. Further complicating the picture, the study of Read, M.L. et al. described a new connection between PTTG1-Binding Factor (PBF), p53, and Mdm2 (the major E3 ubiquitin ligase of p53). They showed PBF’s ability to diminish p53 stability in thyroid cells in an Mdm2-dependent manner without direct PTTG1 involvement [56]. This finding suggests that the relationship between PTTG1 and p53 may be indirect or mediated by other factors. Considering all aspects involved, there is much to uncover regarding the interaction between PTTG1 and p53.

### 3.2. WNT/β-CATENIN Pathway

Wnt/β-catenin signaling regulates key cellular functions and plays an important role in carcinogenesis, including the maintenance of CSC in colorectal cancer (CRC) and abnormal activity in hepatocellular carcinoma (HCC) [57,58]. PTTG1 is also involved in HCC development, and overexpression of PTTG1 was correlated with more advanced tumor size, tumor grade, and TNM stage, and ultimately, poor prognosis [59]. Though the exact mechanism behind PTTG1 activity in HCC is still unknown, evidence suggests a connection between PTTG1 and the Wnt/β-catenin pathway in HCC [58]. PTTG1 is a β-catenin binding protein promoting β-catenin stabilization and facilitating its nuclear accumulation. Consequently, this process leads to excessive activation of Wnt/β-catenin signaling, potentially driving HCC progression [58]. Further research is needed to characterize the relationship between PTTG1 and the Wnt/β-catenin pathway fully. Specifically, future studies should focus on elucidating the precise interaction mechanism between PTTG1 and β-catenin. It is essential to undertake research to identify specific downstream targets of the Wnt/β-catenin pathway activated by PTTG1 in hepatocellular carcinoma (HCC) to elucidate their contributions to tumor progression and poor prognosis. Research to identify specific inhibitors or alternative strategies that may disrupt this interaction and inhibit the growth of hepatocellular carcinoma (HCC) must be conducted to potentially unveil novel therapeutic targets for this challenging cancer and other neoplasms.

### 3.3. TGF-β/SMAD Pathway

The SMAD gene family is an important regulator of the TGF-β signaling pathway, which is known to promote EMT and contribute to the invasion and metastasis of various cancer cells, including BC [60]. PTTG1 may also be involved in the cascade mentioned above, although its precise role and mechanism require further investigation. In BC, PTTG1 levels are significantly higher than in normal tissue, suggesting its potential role in breast cancer pathogenesis [61]. PTTG1 may enhance cell growth in breast cancer by promoting cell proliferation through p27 nuclear exclusion, highlighting a novel mechanism in breast cancer tumorigenesis. Co-immunoprecipitation experiments showed that PTTG11 directly interacts with p27 in BC cells, giving insights into prospective therapeutic targets in BC [61]. Moreover, the study on lung adenocarcinoma cells revealed that PTTG1 protein expression is highly upregulated in lung adenocarcinoma tissues, which is positively correlated with lymphatic invasion of the tumor. PTTG1 is capable of inhibiting TGFβ1/SMAD3 signaling and, in turn, suppressing apoptosis and promoting cancer growth [62]. The findings suggest that PTTG1 could be a potential target for developing immunotherapeutic strategies for lung adenocarcinoma. Further investigation is needed to develop anticancer therapies targeting PTTG1 and TGFβ1/SMAD3 pathways.

### 3.4. PTTG1/c-MYC Pathway

The study of Lin X. et al. showed a dependence between PTTG1 and c-MYC in HCC, as PTTG1 promotes hepatocellular carcinogenesis through the upregulation of c-MYC [59]. However, the influence does not seem to work both ways, since the downregulation of PTTG1 reduces c-MYC expression and inhibits cell proliferation. Nevertheless, inhibition of c-MYC does not affect PTTG1 expression, which shows that regulatory interaction is unidirectional [59]. Importantly, the PTTG1/c-MYC induction is controlled by tumor necrosis factor-α (TNF-α), a key proinflammatory cytokine involved in HCC. This introduces another layer of complexity to the interaction, highlighting the impact of the inflammatory microenvironment [59]. Another signaling loop consisting of PTTG1/c-MYC was observed in CRC, and it also included spindle and kinetochore-associated complex subunit 3 (SKA3) [63]. SKA3 facilitated the translocation of PTTG1 to the nucleus, where PTTG1 serves as the transcriptional activator for the expression of c-MYC [63]. The study of Zhang et al. highlighted that the SKA3/PTTG1-c-MYC signal loop stimulates the proliferation and metastasis of CRC [63].

### 3.5. Current Therapeutic PTTG1-Related Strategies

PTTG1 is a potential therapeutic target in various cancers, with research investigating its role as a biomarker and intervention target. Although more studies and clinical trials are required, initial findings indicate promising therapeutic development options. In pancreatic cancer, PTTG1 overexpression has been described and correlated with a higher histological grade of pancreatic cancer. Thus, PTTG1 may be a valuable marker for the severity of pancreatic cancer [64]. The study consisted of an examination of the PTTG1 messenger RNA (mRNA) expression levels and clinical significance through meta-analysis based on The Cancer Genome Atlas (TCGA) and Gene Expression Omnibus (GEO) databases [64]. Overexpression of PTTG1 was observed in pancreatic cancer tissue, and the higher the histological grade of the cancer, the higher the level of PTTG1 [64]. Certainly, more studies and clinical trials are needed to confirm the utility of PTTG1, but it seems promising. In BC, PTTG1 is estrogen-dependent and correlated with the survival status of patients [65]. Estrogen increased the expression of PTTG1 through binding to the M1 estrogen-responsive site of PTTG1 promoter [65]. What is more, the tamoxifen sensitivities of BCs were also connected to estrogen/PTTG1 influence, as tamoxifen resistance occurs more often in estrogen receptor-positive cancers [65]. The role of PTTG1 in BC became more interesting when it was associated with the anticancer properties of statins [66]. The study of Yin L. et al. showed that simvastatin may suppress BC cell invasion through decreasing PTTG1 mRNA stability, ergo PTTG1 suppression, highlighting a potential therapeutic strategy involving PTTG1 suppression [66]. While promising, several challenges remain in targeting PTTG1 in cancer therapy. It is crucial and challenging to develop strategies that precisely target PTTG1 without disrupting vital cellular processes. It is necessary to develop and validate the efficacy and safety of PTTG1-targeted therapies. That is why further comprehensive studies are required to fully elucidate the therapeutic potential of targeting PTTG1.

## 4. The Interaction of RAC2 and PTTG1 in Cancer

### 4.1. Roles of RAC2 and PTTG1 in Epithelial–Mesenchymal Transition

EMT enables epithelial cells to lose their characteristic markers and adopt mesenchymal traits [67]. This process can be described as the trans-differentiation of stationary epithelial cells into dynamic, motile mesenchymal cells [34]. During EMT, epithelial cells penetrate the basal membrane and migrate to distant locations by initiating precise alterations in their cytoskeletal architecture. EMT plays a crucial role in mammalian growth and development, contributing to wound healing, stem cell maintenance, and tumorigenesis, where cancer cells exploit this mechanism to facilitate metastasis [67,68]. RAC2 and PTTG1 play significant roles in promoting EMT, which gives cancer cells stem-like properties and enhances their metastatic potential.

**Figure 2:**
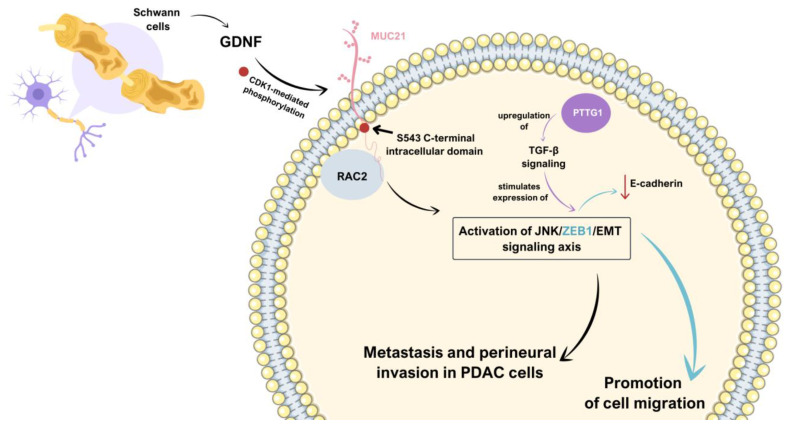
Schematic visualization of the connections between described pathways. GDNF, secreted by Schwann cells, triggers CDK1-mediated phosphorylation of MUC21 at its S543 C-terminal intracellular domain, which enables MUC21 to interact directly with RAC2. That results in JNK/ZEB1/EMT signaling axis initiation, thus promoting metastasis and perineural invasion in PDAC cells. ZEB1, which constitutes an element of the aforementioned signaling axis, directly inhibits E-cadherin expression, which facilitates cell migration. Importantly, the expression of ZEB1 can be promoted by TGF-β signaling, which is upregulated by PTTG1 [69,71].

RAC2’s involvement in EMT is consistent with its known functions in cellular processes such as cytoskeletal actin reconfiguration, cell polarization, migration, and cell–substrate adhesion, all of which are important aspects of the EMT process. Multiple findings of research on cancer cells, described below, reflect this. RAC2’s influence on EMT was found in pancreatic ductal adenocarcinoma (PDAC) [69]. It occurs through a complex signaling cascade that begins when glial cell-derived neurotrophic factor (GDNF), secreted by Schwann cells, triggers CDK1-mediated phosphorylation of mucin 21 (MUC21) at its S543 C-terminal intracellular domain [69]. MUC21, aside from forming a protective mucous barrier covering epithelial cells, also plays a role in intracellular signaling [70]. The phosphorylation mentioned above enables MUC21 to interact directly with RAC2, leading to both membrane anchoring and activation of RAC2. When activated, RAC2 initiates the JNK/ZEB1/EMT signaling axis, eventually promoting metastasis and perineural invasion in PDAC cells [69]. This pathway represents a novel mechanism linking RAC2 to EMT regulation. It suggests that RAC2 is a crucial intermediary between extracellular GDNF signaling and the cellular EMT program, potentially making it a valuable therapeutic target for preventing PDAC progression. The aforementioned transcription factor ZEB1 was also linked, along with JNK, to the signaling pathway leading to mediation of gemcitabine resistance via down-regulation of its transporter ENT1 [71]. This pathway may play a role in mediating tumor resistance to chemotherapeutic treatments since ZEB1 knockout was shown to increase gemcitabine sensitivity [71]. The activation of ZEB proteins is essential for suppressing E-cadherin expression and facilitating cell migration, and in mouse mammary epithelial cells, ZEB proteins directly inhibit E-cadherin expression independently of the Snail transcription factors [72]. This fact, in combination with the previously described RAC2–ZEB1 relationship, further implies RAC2’s vital position in the EMT process. Moreover, since ZEB protein expression can be promoted by TGF-β signaling [73], which takes part in the PTTG-1-dependent process of EMT (described further), a crossing in pathways of those two proteins appears (Figure 2).

**Figure 3:**
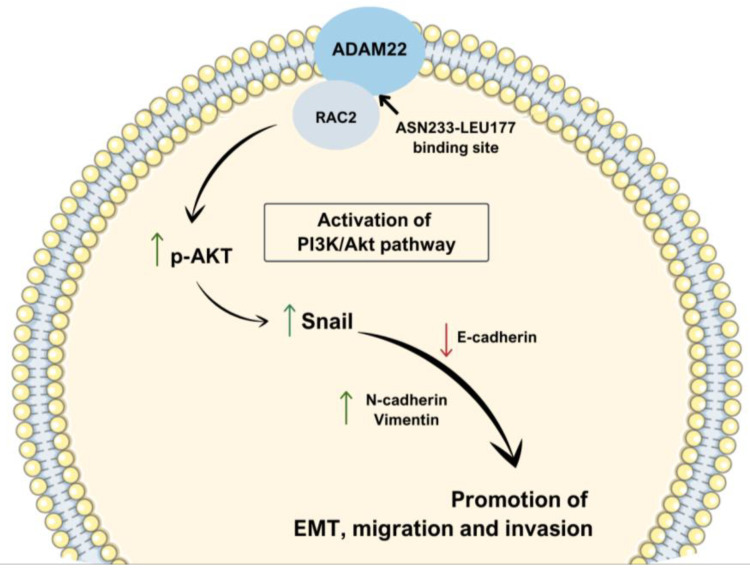
Schematic visualization of the interaction between ADAM22 and RAC2, which results in EMT promotion [79].

Interestingly, when Juvenile Nasopharyngeal Angiofibroma (JNA) cells were examined for gene expression profile in search for regulators of EMT, RAC2 expression was significantly decreased [74]. JNA, despite being a benign tumor, is characterized by its local expansion and destructiveness; therefore, this significant shift in RAC2 expression indicates its potentially diverse role in tumor development [75]. What is more, a study aiming to assess macrophage signature in the context of predicting prognosis in head and neck squamous cell carcinoma by employing a weighted gene co-expression network pinpointed the RAC2 gene among eight other genes to the high-risk group that was characterized by lower overall survival at the patient level and immune evasion capacity along with EMT tendency at the cellular level [76]. Nasopharyngeal carcinoma is challenging to treat due to its marked tendency for metastasis and diffuse spread since it exhibits the greatest propensity for regional lymph node metastasis compared to other types of head and neck squamous cell carcinoma [77,78]. RAC2 appears to play an important role in mediating EMT in this type of cancer through its interaction with ADAM22 and the PI3K/Akt signaling pathway [79]. The research discovered that when RAC2 was inhibited, it reversed the EMT process that had been previously enhanced by ADAM22 overexpression, as evidenced by higher expression levels of the epithelial marker E-cadherin and reduced levels of the mesenchymal markers N-cadherin and vimentin [79]. RAC2 directly interacts with ADAM22 through a noncovalent bond at the ASN233-LEU177 binding site, and this interaction appears to be crucial for activating the discussed pathway, which in turn promotes EMT [79]. RAC2 knock-down in cells overexpressing ADAM22 results in the reduction in cell migration and invasion capabilities but also suppression of the EMT phenotype by altering the expression of EMT markers [79]. This suggests that RAC2 serves as a critical mediator between ADAM22 and the EMT process, primarily through its role in the signaling cascade (Figure 3) [80,81,82].

PTTG1 plays a crucial role in inducing EMT in ovarian cancer [83]. It upregulates TGF-β expression and secretion, which in turn increases the expression of key EMT transcription factors, including Twist, Snail, and Slug [83]. These transcription factors then act to repress E-cadherin expression, a hallmark of EMT [84,85]. The loss of E-cadherin serves as a critical early event in the trans-differentiation of epithelial cells into a mesenchymal phenotype, which facilitates the invasion of tumor epithelial cells into surrounding tissues [86]. PTTG1 overexpression induces the morphological changes characteristic of EMT, with cells transforming from a flat and elongated epithelial shape to a round and spherical form with lamellipodia and filopodia, indicating increased invasive potential [83]. PTTG1 overexpression leads to decreased E-cadherin and increased vimentin expression, and PTTG1 knockdown using siRNA causes reduced expression of Twist, Snail, and Slug, increased E-cadherin, and decreased vimentin (a mesenchymal marker) [83]. Researchers confirmed that these effects were mediated through TGF-β by showing that blocking TGF-β with a neutralizing antibody prevented the PTTG1-induced changes in EMT markers [83]. These findings further imply that PTTG1 plays an important role in tumor progression and metastasis. Another study supports this thesis by establishing that PTTG1 and TGF-β signaling appear to drive cellular behaviors characteristic of EMT in glioblastoma [13]. The molecular mechanism appears to operate through PTTG1’s ability to maintain or activate TGF-β signaling, which in turn engages the PI3K-AKT-mTOR pathway [13]. This is evidenced by the observation that PTTG1 knockdown suppresses the activity of the discussed pathway, suggesting that PTTG1 normally functions upstream of TGF-β to promote these EMT-like features [13]. When PTTG1 is overexpressed, as observed in GBM patients, it likely amplifies this signaling cascade, leading to enhanced migration and invasion through TGF-β-dependent mechanisms [13]. However, the precise molecular interactions between PTTG1 and TGF-β signaling components would require further investigation to explain the EMT regulatory mechanism fully. What is more, investigations into HCC have revealed a regulatory axis governing EMT through PTTG3P- PTTG1 mediated signaling [12]. PTTG3P upregulates PTTG1 expression and, through this mechanism, activates the PI3K/AKT signaling pathway, which subsequently influences EMT-related factors Snail and Slug while simultaneously decreasing the expression of the E-cadherin [12]. Through the PI3K/AKT signaling pathway, PTTG1 appears to be an integral component in the complex network of factors controlling EMT, ultimately contributing to the metastatic potential of hepatocellular carcinoma cells [12]. Similarly, PTTG1 was found to promote EMT in sinonasal squamous cell carcinoma (SNSCC) [87]. PTTG1 overexpression leads to decreased expression of E-cadherin and elevated levels of N-cadherin and Vimentin [87]. The relationship between PTTG1 and EMT was revealed through experiments where PTTG1 overexpression reversed the EMT-suppressing effects of microRNA miR-362-3p, which normally inhibits EMT by targeting PTTG1 [87,88]. Numerous studies indicate that miR-362-3p may play a suppressive role in human malignancies, such as BC, glioma, oral-squamous-cell, and ovarian cancer [89,90,91,92,93].

### 4.2. The Connection Between RAC2, PTTG1, and NF-κB Signaling

The interaction between PTTG1 and nuclear factor kappa-light-chain-enhancer of B cells (NF-κB) signaling represents a critical axis in cancer development and progression across multiple tumor types [91,92,93]. Recent studies indicate that PTTG1 exerts its oncogenic effects through modulation of the NF-κB signaling pathway, with experimental evidence showing that PTTG1 knockdown leads to significant suppression of tumor progression, specifically through inactivation of the NF-κB pathway [91]. This regulatory relationship appears to be mediated in part through FOXM1, which directly binds to the PTTG1 promoter region and coordinates the expression of key NF-κB pathway genes, including Ikbkb, Nfkb1, Nfkb2, and Rela [92]. Mechanistically, deletion of Foxm1 prevents K-Ras-mediated activation of NF-κB pathway components while simultaneously suppressing PTTG1 expression. In contrast, transgenic overexpression of activated FOXM1 is sufficient to induce both PTTG1 and NF-κB pathway genes. These findings suggest a coordinated regulation that is essential for tumorigenesis, as disruption of this pathway through either PTTG1 knockdown or Foxm1 deletion prevents tumor formation. Interestingly, a cross-species comparative genomic analysis identified PTTG1 as one of 16 evolutionarily conserved genes crucial for tumor progression, with functional network analysis revealing its role in cell cycle regulation, DNA repair, and mitotic processes [93]. The findings establish PTTG1 as both a critical upstream regulator and downstream target within the NF-κB signaling network in cancer progression, highlighting its potential as a therapeutic target. RAC2 emerges as a mediator of NF-κB activation in B-cell malignancies resistant to Bruton tyrosine kinase (BTK) inhibition [94]. In ABC DLBCL cells with ibrutinib resistance, RAC2 appears to substitute for BTK in activating phospholipase Cγ2 (PLCγ2), thereby maintaining NF-κB signaling and cell survival [94]. This enhanced RAC2-PLCγ2 interaction was also observed in chronic lymphocytic leukemia cells from patients with treatment-resistant or progressive disease on BTK inhibitor treatment [94]. The RAC2-dependent signaling circuit is partially mediated through TCF4-dependent transcriptional regulation and can be targeted using RAC1/2-specific small-molecule inhibitors such as NSC-23766 and EHT1864 [95]. The mechanism represents an epigenetic rather than genetic form of resistance. Functionally, RAC2 knockdown in ibrutinib-resistant cells led to decreased expression of NF-κB-regulated genes and reduced cell survival, highlighting RAC2’s essential role in maintaining oncogenic NF-κB signaling BTK inhibitor resistance [94]. Interestingly, neutrophils lacking RAC2 demonstrated markedly reduced production of NF-κB-dependent inflammatory chemokines MIP-1α and MIP-2 in response to bacterial stimulation, suggesting RAC2 functions upstream of NF-κB-mediated inflammatory gene expression in these cells [96]. The alternative pathway for pro-survival NF-κB activation suggests that targeting RAC2 could be a promising therapeutic strategy for overcoming BTK inhibitor resistance in B-cell malignancies and inflammatory processes. RAC2’s crucial role in regulating NF-κB signaling was established in bone marrow-derived macrophages (BMDM) [97]. Experiments conducted in RAC2-deficient mice showed that RAC2 is essential for proper NF-κB activation through regulating IκB degradation [97]. In RAC2-null BMDMs, there were higher baseline levels of IκB and significantly decreased IκB degradation in response to Lipopolysaccharide (LPS) exposure compared to wild-type cells, indicating impaired NF-κB activation. While RAC2-deficient cells showed a compensatory increase in RAC1 expression, they exhibited reduced levels of biologically active GTP-bound RAC following LPS stimulation, suggesting that RAC2 is the preferred substrate for activation in this pathway [97]. This RAC2-dependent regulation of NF-κB signaling was found to influence downstream inflammatory mediators, particularly cyclooxygenase-2 (COX-2) expression, demonstrating RAC2’s importance in inflammatory signaling cascades. In the hematopoietic system, RAC2 was shown to be activated by the GDP/GTP nucleotide exchange factor Vav1, which plays a role in immune cell signaling pathways leading to NF-κB activation [98]. The activation of RAC2 occurs downstream of Vav1, which becomes tyrosine phosphorylated following receptor stimulation in hematopoietic cells [99]. This signaling cascade involves the coordination of multiple pathways, including the activation of PLCγ, which is regulated through both PI3K-dependent and independent mechanisms [98]. The Vav1-RAC2 axis contributes to NF-κB activation through its integration with calcium signaling and ERK pathways, ultimately affecting transcriptional responses [98]. This signaling network regulates cellular processes ranging from proliferation to cytokine production in immune cells, and dysregulated expression of Vav1 was found in cancer cells [99]. Understanding this pathway is important as it represents a potential therapeutic target in diseases where aberrant NF-κB signaling plays a pathogenic role. Emerging evidence indicates that Vav1 exerts functions beyond its guanine nucleotide exchange activity, including regulation of signaling pathways such as JNK, ERK, and NFAT [98,100]. These effects are likely facilitated by Vav1’s interactions with various proteins through its additional modular domains. To conclude, PTTG1 and RAC2 are critical regulators of cancer progression and promising targets in anticancer therapy due to their roles in EMT, signaling pathways like TGF-β, PI3K/AKT, and NF-κB, as well as their influence on immune evasion and therapy resistance. PTTG1 enhances EMT by upregulating transcription factors such as Snail and Twist via TGF-β signaling, suppressing E-cadherin, and promoting invasiveness and metastasis while also modulating NF-κB signaling and tumor cell survival, particularly in aggressive cancers like glioblastoma and HCC. RAC2 contributes to EMT and metastasis by mediating cytoskeletal reorganization and activating pro-tumor pathways like PI3K/AKT and JNK/ZEB1. It is implicated in resistance to therapies, such as BTK inhibitors, through NF-κB activation and immune modulation (Table 1).

Targeting these proteins, individually or together, offers significant potential to inhibit EMT, reduce metastasis, and overcome drug resistance, paving the way for more effective cancer therapies (Table 2).

#### Interaction Between RAC2 and PTTG1 in Cancer Biology

Although direct molecular interactions between RAC2 and PTTG1 have not been conclusively established, existing evidence indicates that these proteins co-regulate critical cancer-related pathways, including epithelial–mesenchymal transition (EMT), immune evasion, and tumor progression. Both RAC2 and PTTG1 contribute to the activation of the NF-κB and PI3K/AKT signaling pathways, which are essential for cancer cell survival, invasion, and therapy resistance [12,19]. For instance, RAC2 is known to modulate NF-κB signaling by interacting with phospholipase Cγ2 in treatment-resistant B-cell malignancies, thereby promoting inflammatory and survival pathways. Similarly, PTTG1 has been shown to influence NF-κB activation through its regulatory effects on FOXM1 expression, linking its activity to tumor progression and immune system modulation [19]. In addition to these shared pathways, both proteins participate in modulating EMT through their influence on transcription factors like Snail, Twist, and ZEB1, which regulate E-cadherin expression and promote metastatic potential [12]. These convergent functions suggest a potential functional interplay, even if the exact molecular relationship remains unclear. Further research, including co-immunoprecipitation assays and pathway-specific analyses, is needed to clarify whether RAC2 and PTTG1 interact directly or operate through distinct but convergent signaling mechanisms.

## 5. Angiogenesis and Tumor Microenvironment (TME)

Hypoxia is recognized as a factor that promotes CSC survival, self-renewal, and proliferation [101]. It is achieved mainly through changes in gene expression by hypoxia-inducible factors (HIFs) [102]. HIF-1 has attracted significant scientific attention, as one of its two subunits, subunit α, is regulated by oxygen concentration, which leads to numerous hypoxia-induced effects, especially in the TME [103]. Apart from the negative regulation by oxygen-dependent degradation, the activity of HIF-1α in CSC is also positively regulated by the PI3K-AKT pathway. In a study by Cui et al., silencing PTTG1 suppressed the activity of the TGF-β/PI3K-AKT-mTOR pathway [13]. Additionally, PI3K seems to enable the production of ROS in BCR-ABL1+ leukemic cells. RAC in a positive feedback loop with PI3K contributes to the production of ROS [104]. Coming from both PTTG1 and RAC, ROS, on the other hand, could facilitate HIF-1α accumulation. In CSCs, HIF-1α would also then activate PDK1, which acts as a superfluous ROS scavenger [105]. Such a function is beneficial because of the low ROS content maintained in CSCs, which, if raised, could decrease their clonogenicity and result in radio sensitization [105,106]. MicroRNA-186 (miR-186) has a regulatory role across different cancer types [107]. It has been discovered that miR-186 binds directly to HIF-1α in gastric adenocarcinoma, inhibiting its protein production [108]. Similarly, miR-186 targets HIF-1α in other neoplasms and downregulates aerobic glycolysis [109,110]. Furthermore, the miR-186 role includes suppression of the PTTG1-mediated oncogenic effects. However, overexpression of PTTG1 could partially abolish the suppressive function of miR-186, as observed by Xiao et al. [109]. Therefore, high PTTG1 expression could indirectly enable the HIF-1α pathway and promote CSCs maintenance, yet this needs more research to clarify. CSCs are known to promote angiogenesis, creating a vascular niche, which contributes to their maintenance and further proliferation, as well as advancing metastases [111]. Angiogenesis is influenced by numerous factors, among which vascular endothelial growth factor (VEGF) stands out [112]. Besides its role in angiogenesis, VEGF maintains CSCs stemness, rendering it important in CSCs TME [113]. Its activity is modulated by, among others, HIF-1α, which remarkably induces VEGF expression [114,115]. As illustrated in the preceding paragraph, RAC2 and PTTG1, through the mechanism of HIF-1α, may consequently play a role in enhancing angiogenesis. In multiple papers, ROS boosted VEGF release, just as inhibition of ROS signaling led to decreased VEGF expression [115,116,117,118]. It seems to be another angiogenic pathway for RAC2 and PTTG1. PTTG1 knockdown significantly reduced the protein levels of different VEGF as well as p-PI3K/PI3K, p-AKT/AKT, and p-eNOS/eNOS, which are reported to play a fundamental role in angiogenesis and vascular endothelial barrier reconstruction too [13,119].

## 6. Immune Evasion

In the TME, there is an interplay between CSCs and immune cells, modulating immune responses through a combination of distinct mechanisms [120]. The work of Tsuchiya et al. from 2021 concludes that the immune evasion property, rather than the tumor-initiating property, is a more fundamental feature of CSCs [121]. Overproduced ROS, and ROS in conjunction with nitrogen species (RNS), activates inflammatory pathways such as JAK/STAT and NF-κB signaling, which leads to inhibition of antitumor immune response [122]. Cytotoxic T-cells infiltrating a tumor are pivotal in the antitumor immune response [123]. Elevated levels of ROS can impede the recognition between T cell receptors (TCR) and MHC-peptide complexes, in addition to inducing T cell apoptosis or necrosis, thus ensuring protection for CSCs [122]. Different lymphocytes—regulatory T-cells (Tregs)—present in the TME are immunosuppressive, decreasing tumor-reactive cytotoxic T-cell immunity. ROS also influence Tregs, leading to a more robust immunosuppressive effect [123]. Myeloid-derived suppressor cells (MDSCs) serve as a significant immunosuppressive cell type within the TME. MDSCs can produce ROS, as well as be stimulated by ROS, presenting an inhibitory effect on antigen-specific T cells, B, and NK cells [105]. Furthermore, MDSCs increase the expression of another immunosuppressive molecule, tumor-programmed death-ligand 1 (PD-L1), via the PI3K-AKT-mTOR pathway [124].

In the TME, HIF-1α recruits and increases the function of suppressive cells, including tumor-associated macrophages and MDSCs. HIF-1α also induces the expression of PD-L1 on cancer cells and cytotoxic T-lymphocyte-associated protein 4 (CTLA4) on CD8+ T-cells, which reduces T cell-mediated antitumor responses [114]. It appears that RAC2 and PTTG1, through the promotion of ROS and HIF-1α, could facilitate immune evasion; however, further research on the matter is needed. As mentioned in previous paragraphs, RAC2 induces PI3K activation, which fosters the development, maturation, homing, and function of NK cells, an important factor of antitumor defense [125]. A recent study has identified that PTTG1 upregulates the expression of particular immune checkpoint genes, facilitating tumor cells to evade immune surveillance. High expression of PTTG1 is correlated with enhanced immune cell infiltration in most tumors. Furthermore, expression levels of PTTG1 correlate to various immune cell types infiltrating tumor niches, including T cells, B cells, neutrophils, and macrophages. These findings show PTTG1 as a possible therapeutic target and potential guide for immunotherapy, as it seems to modulate TME. Wang and Liu laid the groundwork for future investigations into the underlying mechanisms [126]. There is a need to evaluate the potential of PTTG1 as a prognostic biomarker for response to immune checkpoint inhibitor therapies as well as combination treatment strategies.

## 7. Apoptosis and Autophagy Pathways

**Figure 4:**
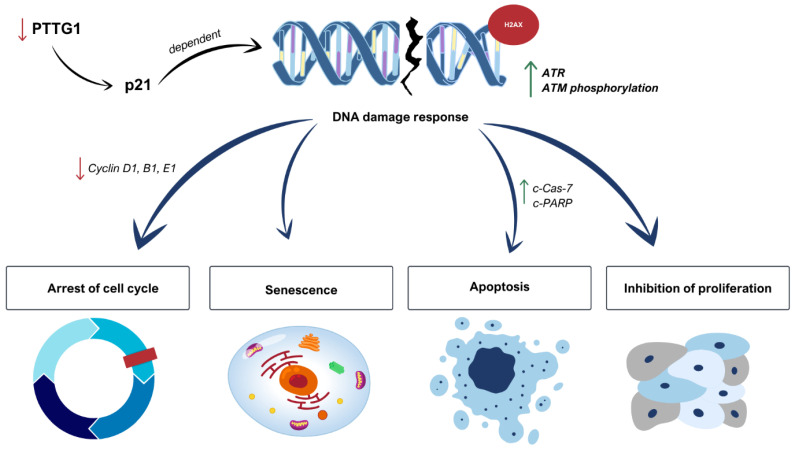
Schematic visualization of the way in which PTTG1 downregulation triggers p21-mediated DNA damage response leading to cell cycle arrest, senescence, apoptosis, and proliferation inhibition [135].

Apoptotic signaling pathways, which control cells’ life cycle, are downregulated in CSCs, contributing to their survival and prevailing resistance to therapies [127]. CSCs are known to both activate antiapoptotic pathways and inactivate pro-apoptotic pathways, in which RAC2 and PTTG1 [128,129]. The available studies have shown that levels of RAC2 and antiapoptotic Bcl-2 family proteins are interconnected [128,130]. Mizukawa et al. performed a knockdown of RAC2, which decreased expression of Bcl-2 and Bcl-xL and induced apoptosis [128]. It has been frequently reported that PI3K regulated the G1 cell cycle in different cancers and that inhibition of PI3K/Akt/mTOR signaling induced apoptosis [131,132]. RAC2 and PTTG1 silencing downregulates PI3K-Akt, therefore deactivating apoptosis evasion, which has been better researched in PTTG1-silencing [13,133]. PTTG1 is widely considered to regulate apoptosis in various neoplasms [13,129,134,135,136]. A 2002 study by Bernal et al. demonstrated that PTTG1 protein inhibits the ability of p53 to induce cell death [137]. Since then, different papers evidenced either decreased p53 expression due to PTTG1 knockdown [135], or high tumoral PTTG1 and PTTG1-Binding Factor (PBF/PTTG1IP), resulting in an aberration of p53-dependent signaling [138]. A 2024 study by Park et al. determined that PTTG1 knockdown in oral squamous cell carcinoma (OSCC) promotes DNA damage in a p21-dependent manner, leading to an increase in phosphorylation of ataxia telangiectasia mutated (ATM) and ataxia telangiectasia and Rad3-related (ATR) proteins, which results in the formation of γH2AX, known to play a critical role in inducing apoptosis in cancer cells. How PTTG1 regulates the p21-dependent pathway requires further investigation. Moreover, in the described study, cyclins D1, B1, and E1, responsible for cell proliferation, cell division, and DNA replication, respectively, were significantly downregulated. In contrast, the expression of apoptotic markers such as cleaved Caspase-7 (c-Cas-7) and cleaved poly (ADP-ribose) polymerase (c-PARP) was significantly increased. In summary, Park et al. determined that PTTG1 level in OSCC induces cycle arrest, DNA damage, and cell death mediated by p21 (Figure 4) [135]. Finally, PTTG1-knockdown suppresses the NF-κB signaling pathway, which performs an antiapoptotic role [139,140].

Autophagy, on the other hand, is a multistep lysosomal degradation process that degrades and recycles cytoplasmic organelles or components to maintain cellular homeostasis in the face of new environmental stressors [139,140]. CSCs are believed to utilize autophagy as a pro-survival mechanism to maintain a dormant-like stage, providing them resistance against therapeutic stresses such as chemotherapy and radiotherapy [140]. ROS levels are one of the factors that activate autophagy in stress conditions, both directly and indirectly. Similarly, HIF-1α has been observed to trigger autophagy in BC cells [123]. In hepatocellular carcinoma cells, autophagy can also be induced by suppressing the PI3K/Akt/mTOR pathway [132]. Extensive research is needed to determine whether PTTG1 could regulate autophagy through ROS, HIF-1α, and PI3K/Akt/mTOR pathways in CSCs. RAC3 knockdown, another member of the Rho GTPase subfamily, led to increased autophagy via the same pathway in bladder cancer cells [141]. Interestingly, in another study, the knockdown of RAC2 did not lead to autophagy [94]. Nevertheless, there is limited understanding regarding the impact of RAC2 on autophagy within neoplasms.

## 8. Summary

The roles of RAC2 and PTTG1 are critical in the molecular pathways underlying cancer biology, with both molecules contributing significantly to tumor initiation, progression, metastasis, and resistance to therapies. These proteins influence essential signaling pathways that control cancer cell behavior and the TME. RAC2 controls cytoskeleton reorganization, cancer cell migration, invasion, and EMT. It regulates ROS production through NADPH oxidase and contributes to genomic instability and metabolic shifts in CSCs. The generation of ROS enhances immune evasion by impairing T-cell function and promoting immunosuppressive conditions that support cancer survival, including resistance to therapies like ionizing radiation. PTTG1 participates in various cellular processes, including cell cycle regulation, apoptosis suppression, and EMT. It drives EMT through TGF-β and Wnt/β-catenin signaling, promoting the expression of transcription factors like Snail, Slug, and Twist, which suppress E-cadherin and enable metastasis.

PTTG1 modulates c-MYC expression and interacts with NF-κB signaling to support tumor cell proliferation and protect against apoptosis. Additionally, PTTG1 influences angiogenesis by regulating VEGF expression through HIF-1α and the PI3K/AKT/mTOR pathway, thereby enhancing CSC survival and tumor progression. RAC2 and PTTG1 share common pathways, including their roles in EMT, ROS regulation, and activation of the PI3K/AKT/mTOR axis, which promote metastatic capabilities, immune evasion, and therapy resistance. By facilitating immune suppression through the activation of inflammatory pathways such as NF-κB, both proteins contribute to a tumor microenvironment that is less responsive to immune surveillance and therapeutic interventions. These molecules also affect CSC properties by enhancing stemness, self-renewal, and drug resistance. The suppression of pro-apoptotic signals by PTTG1 and the regulation of ROS by RAC2 enable CSCs to survive in adverse conditions like chemotherapy and radiation therapy. Furthermore, both RAC2 and PTTG1 promote the formation of a vascular niche by upregulating VEGF and contributing to angiogenesis, which further supports tumor growth and metastasis.

The clinical significance of RAC2 and PTTG1 is further emphasized by highlighting their roles as potential biomarkers and therapeutic targets. RAC2 has been identified as a prognostic biomarker in clear-cell renal carcinoma [51], while PTTG1 has shown potential as a prognostic indicator in hepatocellular carcinoma due to its role in promoting β-catenin stabilization [58]. There are promising therapeutic developments, such as the small-molecule inhibitors NSC-23766 and EHT1864, which target RAC2-mediated signaling pathways and are currently being explored for their clinical efficacy [25]. While significant progress has been made in understanding the roles of RAC2 and PTTG1 in cancer biology, several knowledge gaps remain. For instance, the involvement of RAC2 in autophagy, particularly its potential regulatory functions via PI3K/AKT/mTOR and ROS pathways, has not been fully elucidated [23]. Existing research indicates that RAC3 can influence autophagy in bladder cancer cells, but whether RAC2 exhibits a similar mechanism in other malignancies requires further investigation [25]. Additionally, the tissue-specific functions of PTTG1 and its differential expression patterns across cancers such as hepatocellular carcinoma and breast cancer warrant more extensive study [12]. Future research should focus on (1) exploring the potential of RAC2 inhibitors like NSC-23766 and EHT1864 in diverse cancer models, (2) assessing the prognostic significance of PTTG1 expression in clinical cohorts, and (3) conducting mechanistic studies to determine whether RAC2 and PTTG1 interact directly or operate independently through parallel pathways. Understanding these mechanisms could provide more targeted therapies and better prognostic tools for cancer patients.

The roles of RAC2 and PTTG1 in cancer biology underscore their potential as valuable prognostic markers and therapeutic targets. Overexpression of both proteins correlates with poor prognosis in various cancers, including pancreatic ductal adenocarcinoma, nasopharyngeal carcinoma, glioblastoma, and renal cell carcinoma. Targeting their signaling pathways could provide promising strategies for improving cancer treatment outcomes and overcoming therapeutic resistance.

## Figures and Tables

**Table 1:** Summary of key pathways affected by RAC2 and/or PTTG1, highlighting their involvement in cancer progression. These pathways are critical for processes such as cell migration, immune evasion, metabolic adaptation, and therapy resistance. The listed pathways have been implicated in various cancers, including leukemia, breast cancer, and hepatocellular carcinoma. Understanding these interactions can guide future research into targeted therapies, particularly in relation to the PI3K/AKT/mTOR and NF-κB pathways, which are central to cancer cell survival and immune modulations.

Pathway	Protein	Function	References
Actin cytoskeleton reorganization	RAC2	Facilitates cell movement and migration via actin polymerization.	[21,22,23]
ROS production	RAC2	Drives oxidative stress, impacting differentiation of cancer cells; influences radiation resistance.	[29,33]
NOTCH	RAC2	Function is context-dependent, with RAC2 acting as a negative NOTCH regulator in normal T cell development and as a downstream effector of NOTCH signaling in NOTCH1-mutated chronic lymphocytic leukemia cells.	[39,40]
ERK-MAPK	RAC2	Regulates antioxidant capacity, and this takes part in creating a radiation resistance profile.	[34]
NF-κB	RAC2, PTTG1	Mediates immune evasion and regulates inflammatory responses	[98,99,101]
PI3K/AKT/mTOR	RAC2, PTTG1	Supports cancer progression and cell survival	[87,88]
Wnt/β-catenin	PTTG1	Stabilizes β-catenin and facilitates its nuclear accumulation.	[59,60]
TGF-β/SMAD	PTTG1	Suppresses apoptosis and promotes cancer growth.	[62]
PTTG1/c-MYC pathway	PTTG1	Promotes carcinogenesis, proliferation, and metastasis through c-MYC upregulation.	[93,96]

**Table 2:** Emerging therapies targeting RAC2 and PTTG1 in various cancer types. The listed approaches include small-molecule inhibitors, RNA interference, and pathway-specific interventions. These therapies aim to disrupt cancer progression by modulating key signaling pathways such as NF-κB and PI3K/AKT/mTOR. The table highlights the clinical relevance of these targets in cancers like clear cell renal carcinoma (ccRCC), breast cancer (BC), hepatocellular carcinoma (HCC), and pancreatic cancer. The observed outcomes demonstrate the potential of these treatments to inhibit cell proliferation, suppress EMT, and overcome drug resistance, providing a foundation for future translational research.

Therapeutic Target	Disease/Context	Approach	Key Outcome	References
RAC2	ccRCC, BC	Small-molecule inhibitors NSC-23766, EHT1864	Reduced survival and proliferation of cancer cells	[52,53]
PTTG1	HCC	Downregulation using RNA interference	Cell proliferation inhibition, EMT suppression	[88]
PTTG1	Pancreatic cancer	PTTG1 mRNA expression level examination	Promising marker for pancreatic cancer	[66]
PTTG1	BC	Regulating PTTG1 level, which is increased by estrogen	Potential salvation of tamoxifen resistance problem	[67]
Combined RAC2/PTTG1	Multicancer context	Targeting shared pathways like NF-κB or PI3K/AKT/mTOR	Potential therapeutic synergy	[87,98,99]

## Data Availability

Not applicable.
